# Protective Yeasts Control *V. anguillarum* Pathogenicity and Modulate the Innate Immune Response of Challenged Zebrafish (*Danio rerio*) Larvae

**DOI:** 10.3389/fcimb.2016.00127

**Published:** 2016-10-14

**Authors:** Mario Caruffo, Natalie C. Navarrete, Oscar A. Salgado, Nelly B. Faúndez, Miguel C. Gajardo, Carmen G. Feijóo, Angélica Reyes-Jara, Katherine García, Paola Navarrete

**Affiliations:** ^1^Laboratorio de Microbiología y Probióticos, Instituto de Nutrición y Tecnología de los Alimentos, Universidad de ChileSantiago, Chile; ^2^Departamento de Ciencias Biologicas, Facultad de Ciencias Biologicas, Universidad Andres BelloSantiago, Chile; ^3^Instituto de Ciencias Biomédicas, Universidad Autónoma de ChileSantiago, Chile

**Keywords:** yeast probiotic, *V. anguillarum*, innate immune system, zebrafish, protective mechanisms

## Abstract

We investigated mechanisms involved in the protection of zebrafish (*Danio rerio*) larvae by two probiotic candidate yeasts, *Debaryomyces hansenii* 97 (Dh97) and *Yarrowia lypolitica* 242 (Yl242), against a *Vibrio anguillarum* challenge. We determined the effect of different yeast concentrations (10^4^–10^7^ CFU/mL) to: (i) protect larvae from the challenge, (ii) reduce the *in vivo* pathogen concentration and (iii) modulate the innate immune response of the host. To evaluate the role of zebrafish microbiota in protection, the experiments were performed in conventionally raised and germ-free larvae. *In vitro* co-aggregation assays were performed to determine a direct yeast-pathogen interaction. Results showed that both yeasts significantly increased the survival rate of conventionally raised larvae challenged with *V. anguillarum*. The concentration of yeasts in larvae tended to increase with yeast inoculum, which was more pronounced for Dh97. Better protection was observed with Dh97 at a concentration of 10^6^ CFU/mL compared to 10^4^ CFU/mL. In germ-free conditions *V. anguillarum* reached higher concentrations in larvae and provoked significantly more mortality than in conventional conditions, revealing the protective role of the host microbiota. Interestingly, yeasts were equally (Dh97) or more effective (Yl242) in protecting germ-free than conventionally-raised larvae, showing that protection can be exerted only by yeasts and is not necessarily related to modulation of the host microbiota. Although none of the yeasts co-aggregated with *V. anguillarum*, they were able to reduce its proliferation in conventionally raised larvae, reduce initial pathogen concentration in germ-free larvae and prevent the upregulation of key components of the inflammatory/anti-inflammatory response (*il1b, tnfa, c3, mpx*, and *il10*, respectively). These results show that protection by yeasts of zebrafish larvae challenged with *V. anguillarum* relates to an *in vivo* anti-pathogen effect, the modulation of the innate immune system, and suggests that yeasts avoid the host-pathogen interaction through mechanisms independent of co-aggregation. This study shows, for the first time, the protective role of zebrafish microbiota against *V. anguillarum* infection, and reveals mechanisms involved in protection by two non-*Saccharomyces* yeasts against this pathogen.

## Introduction

A wide range of potentially probiotic bacteria have been tested in aquaculture to control infectious fish diseases (Hai, 2015). In contrast, few studies have addressed the protective effects of yeasts or the mechanisms involved in protection (Gatesoupe, 2007; Navarrete and Tovar-Ramírez, 2014). In those studies, modulation of the host immune system has been posited as a possible mechanism involved in the protection of fish against pathogens. An enhanced immune response, reflected by a higher IgM level, was observed in recovering juvenile leopard groupers (*Mycteroperca rosacea*) fed with *Debaryomyces hansenii* (CBS8339) and infected with the dinoflagellate *Amyloodinium ocellatum* (Reyes-Becerril et al., 2008). Olive flounder (*Paralichthys olivaceus*) infected with *Uronema marinum* and fed with the baker's yeast *Saccharomyces cerevisiae* (KCCM 11201) showed a significant increase in superoxide anion production and serum lysozyme activity compared to infected and non-yeast-fed fish (Harikrishnan et al., 2011). Similarly, *Oreochromis niloticus* treated with *S. cerevisiae* (BGY-25®) and infected with different fish pathogens revealed a significant increase in total protein, β and γ globulins compared to controls (Abu-Elala et al., 2013). All these studies have shown an effect on the immune system of the fish, probably due to immunostimulant compounds present in yeasts such as β-glucans, nucleic acids and/or mannanoligosaccharides (Li and Gatlin, 2006; Lokesh et al., 2012).

The control of an infectious disease can also be performed by limiting the growth of the pathogen in the host (Schneider, 2011). Although, there are few studies of antibacterial effects of yeasts compared to bacterial studies, several antagonistic properties against bacteria have been reported and reviewed (Hatoum et al., 2012). These include competition for nutrients, changes in pH, high production of ethanol, stimulation of immunoglobulins and antibacterial compounds by the host and inhibition of the attachment to intestinal cells (Hatoum et al., 2012). However, few *in vivo* studies using yeasts have shown the control of pathogen colonization and reduction of its concentration in broiler and mouse guts (Line et al., 1998; Correa França et al., 2015).

Recent studies demonstrated that a physical interaction (co-aggregation) between the yeast *Saccharomyces boulardii* and *Salmonella enterica* serovar Typhimurium could interfere with bacterial invasion, protecting mice against infection (Martins et al., 2013). Also, several structures of the yeast cell wall such as glucans, mannans, and chitin may play a role in co-aggregation with bacteria (Millsap et al., 1998; Hatoum et al., 2012). Therefore, we hypothesize that this mechanism could also be involved in fish protection by yeasts, interfering with host-pathogen interactions.

The initial contact of pathogens with host occurs in tissues colonized by microbiota such as the gut or skin. This microbiota protects the host from pathogens, in a process referred to as colonization resistance, involving direct and indirect mechanisms and impairing pathogen colonization and invasion (Belkaid and Hand, 2014; Pamer, 2016). The gut microbiota acts as a physical barrier to incoming pathogens by competitive exclusion such as competition for nutrients or attachment sites, production of antimicrobial molecules or stimulation of the host to produce antimicrobial compounds (Sekirov et al., 2010; Belkaid and Hand, 2014). The resistance capacity to colonization of the host microbiota against a pathogen can be studied using germ-free animals challenged with microorganisms. However, the few experiments performed in germ-free fish did not show protection against pathogens of the host microbiota (Rendueles et al., 2012; Oyarbide et al., 2015). In the context of fish protection by yeasts, the host microbiota, which plays crucial roles in important physiological processes such as the immune system maturation, has not been explored.

Zebrafish larvae have been used as a model to study interactions between a host and its microbiota or pathogens, and have multiple advantages which include small size, optical transparency of larvae, short generation times, and the possibility to perform *in vivo* analysis, which makes it a powerful platform to study the innate immune response to infection. The central immune molecules of the zebrafish immune system are similar with mammals (Rauta et al., 2012) and innate immunity can be studied in isolation from adaptive immunity, as the zebrafish lacks functional adaptive immunity until at least 3 weeks post-fertilization (Lam et al., 2004). Inflammation is the first biological response of the immune system to infection or irritation, where cytokines such as interleukin 1b and tumor necrosis factor a have an important role in initiating the pro-inflammatory responses once a microorganism enters the host (Bayne and Gerwick, 2001).

We recently reported on the protective effect of 13 different yeast strains isolated from the gut microbiota of healthy wild and reared fish against a *Vibrio anguillarum* challenge in the zebrafish (*Danio rerio*) model (Caruffo et al., 2015). Infected larvae pre-treated with yeasts showed significantly higher survival rate compared to non-treated larvae. In this study we selected two of those yeasts to explore some mechanisms involved in the observed protection. We determined yeast colonization capacity, the modulation of the innate immune response, the *in vivo* anti-*V. anguillarum* effects and co-aggregation with the pathogen. In addition we determined the role of the zebrafish microbiota in larval protection.

## Materials and methods

### Microorganisms and growing conditions

This study included 2 yeast strains previously isolated and identified from the gut of healthy fish (Raggi et al., 2014). *Yarrowia lipolytica* 242 (Yl242) was isolated from a wild yellowtail (*Seriola lalandi*) and *D. hansenii* 97 (Dh97) from a reared rainbow trout (*Oncorhynchus mykiss*). These two non-*Saccharomyces* species were selected due to their high abundance in commercial fish (Raggi et al., 2014), and the 2 yeast strains (Yl242 and Dh97) protected zebrafish larvae from a *Vibrio anguillarum* challenge, increasing its survival percentage (Caruffo et al., 2015). Yeasts were cultured according to Caruffo et al. (2015) in YPD broth (1% yeast extract, Difco, 1% peptone, Difco, 1% glucose, Merck) or YPD agar (YPD broth with 1.4% agar, Difco) supplemented with 0.05% chloramphenicol (Winkler), at 28°C under aerobic conditions. Inoculation of zebrafish larvae was performed with exponential growth cultures of yeast obtained in YPD broth at 28°C for 24 h.

### Maintenance of conventionally raised (CONV-R) larvae

Tab5 embryos (wild type, WT) were maintained and raised according to Hedrera et al. (2013). All embryos were collected by natural spawning, staged according to Kimmel et al. (1995) and raised at 28°C in sterile E3 medium (1% NaCl, 0.17 mM KCl, 0.33 mM CaCl_2_, 0.33 mM MgSO_4_, and 0.00003% methylene blue, Winkler, pH 7.0) in sterile Petri dishes (100 embryos/dish). 75% of the E3 volume was replaced daily with sterile E3 to avoid waste accumulation and oxygen limitation. At 3 dpf (days post-fertilization), larvae were transferred to six-well sterile tissue culture plates (20 larvae/well). Larvae were euthanized with an overdose of tricaine methanesulfonate (4%, MS-222, Sigma-Aldrich).

### Germ-free larvae

Germ-free larvae were generated as previously described (Pham et al., 2008; Milligan-Myhre et al., 2011) with some modifications. Fertilized eggs, obtained by natural breeding, were collected and repeatedly washed in sterile E3 medium. In a UV treated hood, eggs were then washed 2 min with polyvinylpyrrolidone–iodine (PVP-I, 0.1%; MDK) and rinsed with sterile E3. Eggs were then immersed in sodium hypochlorite solution (0.003%) for 20 min, rinsed with sterile E3 and maintained for 4 h in E3 with antibiotics [kanamycin (Winkler) 5 μg/mL; ampicillin (Winkler) 200 μg/mL; amphotericin B (Calbiochem) 250 ng/mL; ceftazidime (Opko) 200 μg/mL and chloramphenicol (Winkler) 20 μg/mL]. The medium was replaced daily by fresh sterile E3 with antibiotics until 2 dpf. From 3 dpf on, larvae were maintained in sterile E3 without antibiotics.

Sterility of larvae and E3 was monitored on day 3 dpf, and until day 9 dpf in non-inoculated larvae, as previously described (Pham et al., 2008; Milligan-Myhre et al., 2011). In brief, 3 larvae were homogenized in 150 μL of sterile phosphate buffer saline (PBS, Winkler; with a 25-gauge needle). One hundred microliter of the homogenate was plated in Trypticase Soy Agar (TSA, BBL), and 50 μL in Trypticase Soy Broth (TSB, BBL). Similarly, the sterility of the E3 medium was verified as previously described. We chose TSA according to a previous recommendation (Milligan-Myhre et al., 2011), and previous results showed that the microbiota of eggs and larvae reared in our facility were best described with this medium incubated aerobically at 28°C (data not shown).

### Protection assays with different concentrations of yeasts

The protection experiments were performed as previously described (Caruffo et al., 2015) with different concentrations of each yeast. Yeast strains were grown at 28°C until the initial exponential phase, pelleted, re-suspended in E3 and transferred to 4 dpf zebrafish larvae at a final concentration ranging from 10^4^ to 10^7^ CFU/mL. Larvae were kept with yeast for 2 h at 28°C then transferred to E3. At 5 dpf, larvae were challenged by immersion with *V. anguillarum* at a concentration of 10^7^ CFU/mL as previously described (Caruffo et al., 2015). The survival rate was recorded daily and monitored for 4 days post-challenge. Control groups were included: a group of larvae inoculated only with (i) yeasts, (ii) *V. anguillarum*, and (iii) non-inoculated larvae. Each group consisted of 60 larvae which were randomly distributed in three wells of a six-well sterile tissue culture plate (in triplicate, 20 larvae/well). Each experiment was independently performed 3 times. The experimental groups are described in Figure S1.

### Yeast and *V. anguillarum* concentrations in zebrafish larvae

To determine the concentrations of yeast and *V. anguillarum* in larvae, 3 larvae of each group were individually homogenized in sterile PBS and serial dilutions were plated in YPD agar supplemented with 0.05% chloramphenicol (Winkler) for yeast count (CFU/larva) or CHROMagar™ Vibrio medium for *V. anguillarum* count.

### Gene expression analysis (RT-qPCR) of innate immune genes

We evaluated the gene expression of some innate immune genes in larvae exposed to different treatments (Table 1). Three pools of 5 larvae per treatment were analyzed. Each pool of larvae was homogenized with a 25-gauge needle and RNA was obtained with the SV Total RNA Isolation System (Promega). cDNAs were synthesized using the ImProm-II™ Reverse Transcription System (Promega) according to the manufacturer's instructions in a TProfessional Thermocycler (Biometra). qPCR was performed in the LightCycler96 (Roche) using FastStart Essential DNA Green Master (Roche) in a 10 μL reaction with a final primer concentration of 500 nM. The primer sequences are detailed in Table 1. The thermal profile used was 95°C 10 min, 40 × (95°C × 30 s, 60°C × 30 s, 72°C × 30 s). Relative expression of RNAm was calculated using 2^−ΔΔCT^ adjusted to primer efficiency (Pfaffl, 2001). beta actin 1 was used as housekeeping gene.

**Table 1:** **Primer sequences used for amplification of specific genes with the RT-qPCR technique**.

**Gene**	**Forward primer (5′-3′)**	**Reverse primer (5′-3′)**	**Amplicon (pb)**	**References**
*b actin1*	TTCTGGTCGTACTACTGGTATTGTG	ATCTTCATCAGGTAGTCTGTCAGGT	144	Guan et al., 2011
*tnfa*	GCGCTTTTCTGAATCCTACG	TGCCCAGTCTGTCTCCTTCT	148	Sepulcre et al., 2009
*il1b*	TGGACTTCGCAGCACAAAATG	GTTCACTTCACGCTCTTGGATG	150	Kanther et al., 2014
*il10*	TCACGTCATGAACGAGATCC	CCTCTTGCATTTCACCATATCC	151	Zhang et al., 2012
*c3*	TGGGAGGCAATAGGCATGA	GCGTAGGATCCATCTGGTTTG	100	Rawls et al., 2004
*mpx*	TCCAAAGCTATGTGGGATGTGA	GTCGTCCGGCAAAACTGAA	90	Rawls et al., 2007

### Co-aggregation assays

#### Macroscopic and microscopic co-aggregation assays

Co-aggregation between the yeasts Dh97 or Yl242, and *V. anguillarum* was performed as previously described (Cisar et al., 1979; Stevens et al., 2015), with modifications. The cell suspensions were adjusted to an O.D. of 4 at 600 nm in co-aggregation buffer (TRIS 0.001M pH8, CaCl_2_ 0.0001M, MgCl_2_ 0.0001M, NaN_3_ 0.02%, and NaCl 0.15 M; Winkler). Equal volumes (200 μL) of *V. anguillarum* and each yeast suspension were mixed in borosilicate tubes (12 × 75 mm, Schott) for at least 5 s in vortex. Visual co-aggregation was scored as previously described (Cisar et al., 1979). Control tubes containing 200 μL of each microorganism and 200 μL co-aggregation buffer were included to check potential auto-aggregation. All suspensions were observed in an optical microscope to observe any microscopic co-aggregation.

#### Spectrophotometric co-aggregation assays

Spectrometric co-aggregation experiments were performed using different media to suspend the microbial cells. Microbial pellets were suspended in 10 mL of PBS (Ogunremi et al., 2015), YPD (Furukawa et al., 2011) and E3, and adjusted to an O.D. of 1.0 at 600 nm. The suspensions of each yeast strain and *V. anguillarum* were mixed in equal volumes (5 mL) for 10 s in vortex. The upper suspension (1 mL) from each test was collected at 1 and 24 h, and O.D. was measured at 600 nm. Control tubes contained 10 mL of each microbial suspension. The percentage of co-aggregation was calculated using the following equation (Ogunremi et al., 2015):

$$\begin{array}{l} \text{Co}-\text{aggregation}\left(\%\right)=\frac{\left(\text{Ax}+\text{Ay}\right)/2-\text{A}\left(\text{x}+\text{y}\right)}{\left(\text{Ax}+\text{Ay}\right)/2}\times 100 \end{array}$$

Ax and Ay represent the O.D. of the two strains in the control tubes, and A(x + y) the O.D. of the mixture. A co-aggregation of >20% was considered positive.

### Statistical analysis

Statistical analysis was performed using the GraphPad Prism 6 software (Graphpad Software, Inc). Survival data were analyzed using the Kaplan-Meier test and group differences were analyzed by the Wilcoxon test, using the Bonferroni correction for multiple comparisons. Differences in mean concentrations of yeasts and *V. anguillarum* were analyzed by Student's *t*-test. The correlation between yeast inoculum and colonization was evaluated by Spearman correlation. The analysis of the RT-qPCR results was calculated relative to the beta actin 1 transcript, and presented as relative expression (2^−ΔΔCt^); differences between groups were analyzed by ANOVA with the Dunnet multiple comparison corrected test. *P* ≤ 0.05 was considered significant.

### Ethical statement

This study was carried out in strict accordance with the recommendations included in the “Guidelines for the care and use of fish in research” and the “Canadian Council on Animal Care's Guide to the Care and Use of Experimental Animals” (Canadian Council on Animal Care, 1989). The protocol was approved by the Committee on the Ethics of Animal Experiments of INTA, University of Chile and FONDECYT (FONDECYT 11110414).

## Results

### *V. anguillarum* challenge

**Figure 1:**
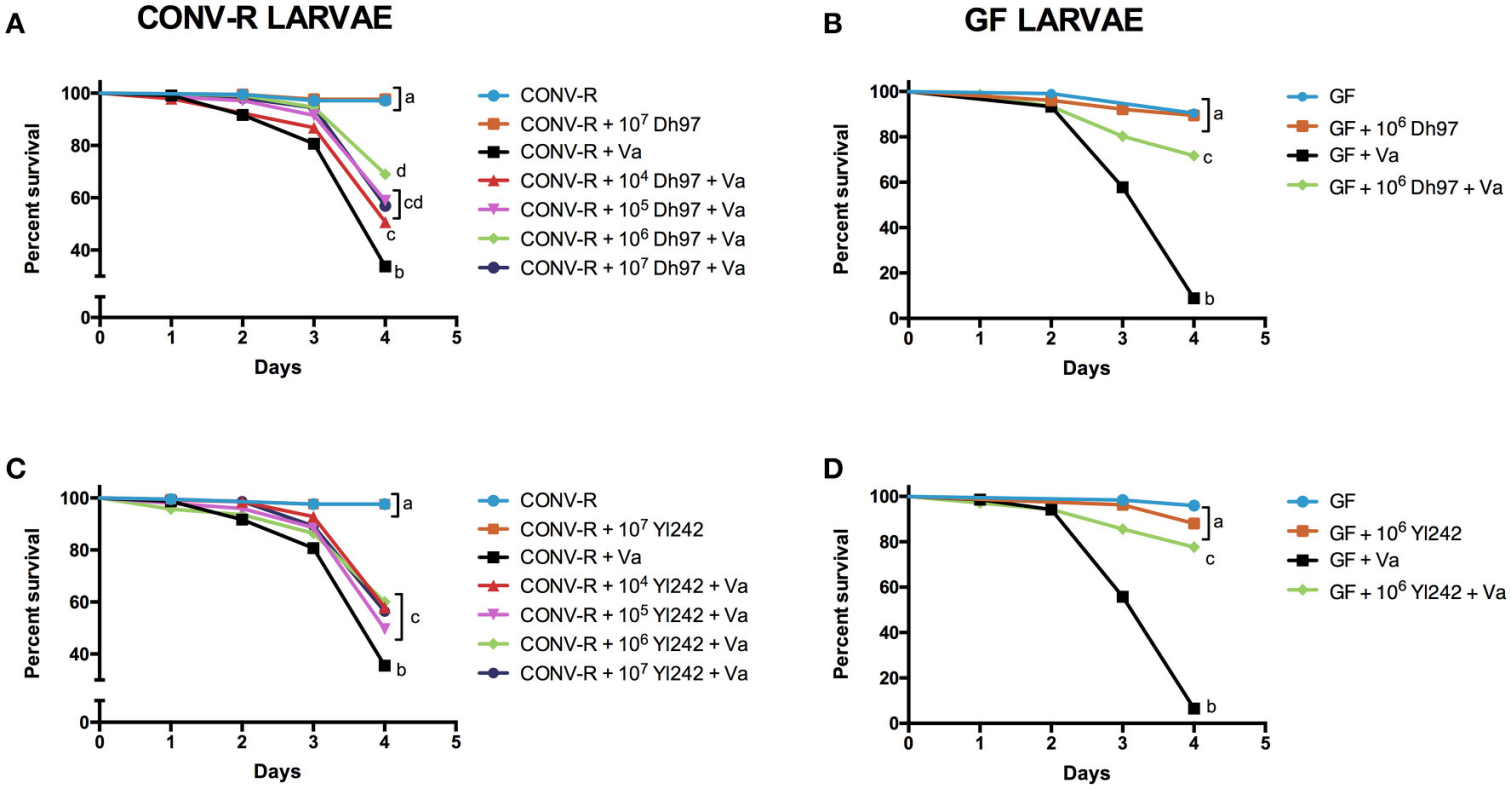
**Protective effect of yeasts ***Debaryomyces hansenii*** (Dh97) and ***Yarrowia lipolytica*** (Yl242) against a ***V. anguillarum*** (Va) challenge in zebrafish larvae**. Larvae were inoculated with different concentration (10^4^–10^7^ CFU/mL) of Dh97 **(A,B)** and Yl242 **(C,D)** on day 4 dpf, and challenged with *V. anguillarum* on day 5 dpf. Survival rate (%) of conventionally raised larvae (CONV-R) **(A,C)**, and germ-free larvae (GF) **(B,D)** at 4 days post- *V. anguillarum* inoculation. The results show the mean ± SD of 3 independent experiments with three replicates each. Different letters indicate statistically significant differences among groups (Kaplan Meier, Wilcoxon *P* < 0.003).

Figure 1 shows the survival rate (%) of zebrafish larvae exposed to different treatments (Figure S1). We observed a significant decrease in the survival rate of the conventionally raised (CONV-R) larvae exposed to the pathogen (Figures 1A,C). To evaluate the effect of zebrafish microbiota on the *V. anguillarum* challenge, we challenged germ-free (GF) larvae with the pathogen. A stronger lethal effect of the pathogen was observed in GF larvae than in CONV-R larvae (*P* < 0.001, unpaired *t*-test; Figures 1B,D).

### Effect of yeast strain inoculum on survival of *V. anguillarum*-challenged larvae

The effect of the yeast inoculum (CFU/mL) on the survival rate (%) of *V. anguillarum*-challenged larvae is shown in Figure 1. We observed that both yeasts, Dh97 and Yl242, significantly protected CONV-R larvae from the *V. anguillarum* challenge (Figures 1A,C). In Dh97 a tendency of dose-dependent protection until 10^6^ CFU/mL was observed, with higher protection of CONV-R larvae pre-treated with 10^6^ CFU/mL compared to 10^4^ CFU/mL (Figure 1A). On the contrary, yeast Yl242, displayed a similar protective effect independent of the concentration used (Figure 1C). Likewise, at 10^5^ and 10^6^ CFU/mL, Dh97 was more effective in protecting CONV-R larvae compared to Yl242 (*P* < 0.05 and *P* < 0.01, respectively; Kaplan-Meier, log-rank post-test).

In GF larvae challenged with *V. anguillarum*, both yeasts were able to increase survival rate significantly at 10^6^ CFU/mL (Figures 1B,D). The effectiveness of yeast Dh97 was similar in GF and CONV-R larvae (*P* = 0.7209, unpaired *t*-test; Figures 1A,B); whereas yeast Yl242 was more effective in protecting GF than CONV-R larvae (*P* < 0.001, unpaired *t*-test; Figures 1C,D).

### Concentration and persistence of yeasts and *V. anguillarum* in CONV-R and GF larvae

To evaluate if the protection of larvae was related to the concentration and persistence of yeast or *V. anguillarum* in larvae, we determined the cultivable count of these microorganisms in larvae. The initial concentration of yeast reached in CONV-R larvae (4 dpf), after 2 h immersion depended on the yeast species and dose (Table 2 and Figure S2). At the same inoculum in E3 medium (CFU/mL), Dh97 reached significantly higher concentrations in CONV-R larvae than Yl242 (*P* < 0.05, unpaired *t*-test). For both yeasts we observed a positive correlation between yeast inoculum and yeast concentration in CONV-R larvae (CFU/larva; *r* = 0.9203, *P* < 0.0001 for Dh97; and *r* = 0.7778, *P* < 0.001 for Yl242, Spearman correlation test; Figure S2). In GF larvae (4 dpf) Dh97 reached similar concentrations as in CONV-R larvae (*P* > 0.05, unpaired *t*-test); whereas Yl242 reached higher concentrations (*P* < 0.05, unpaired *t*-test; Table 2). For all experimental groups, both yeasts persisted in larvae and at 9 dpf reached similar or higher concentrations compared to 4 dpf (Table 2).

**Table 2:** **Initial counts (4 dpf) and persistence (9 dpf) of yeasts in CONV-R and germ-free (GF) larvae**.

	**Yeast concentration (log**_10_**CFU/larva)**							
**Yeast dose**	**CONV-R** + **Dh97**		**CONV-R** + **Dh97** + **Va**		**GF** + **Dh97**		**GF** + **Dh97** + **Va**	
**log_10_CFU/mL**	**4 dpf**	**9 dpf**	**4 dpf**	**9 dpf**	**4 dpf**	**9 dpf**	**4 dpf**	**9 dpf**
ni	<	<	<	<	<	<	<	<
4	2.4 ± 0.2	2.5 ± 0.5	2.4 ± 0.2	3.8 ± 0.0^**^	−	−	−	−
5	3.3 ± 0.1	3.4 ± 0.2	3.3 ± 0.1	3.2 ± 0.3	−	−	−	−
6	3.2 ± 0.1	3.6 ± 0.3	3.2 ± 0.1	2.8 ± 0.0^*^	3.0 ± 0.1	4,2 ± 0.2^***^	3,0 ± 0.1	3.4 ± 0.4
7	3.5 ± 0.1	3.8 ± 0.3	3.5 ± 0.1	3.8 ± 0.1	−	−	−	−
	**CONV-R** + **Yl242**		**CONV-R** + **Yl242** + **Va**		**GF** + **Yl242**		**GF** + **Yl242** + **Va**	
	**4 dpf**	**9 dpf**	**4 dpf**	**9 dpf**	**4 dpf**	**9 dpf**	**4 dpf**	**9 dpf**
ni	<	<	<	<	<	<	<	<
4	1.9 ± 0.1	3.0 ± 0.0^**^	1.9 ± 0.1	2.8 ± 0.2^*^	−	−	−	−
5	2.1 ± 0.0	3.3 ± 0.0^**^	2.1 ± 0.0	3.0 ± 0.0^**^	−	−	−	−
6	2.3 ± 0.1	3.4 ± 0.1^**^	2.3 ± 0.1	3.2 ± 0.2^*^	3.1 ± 0.4	3.5 ± 0.2	3.5 ± 0.1	3.8 ± 0.0^**^
7	2.3 ± 0.0	3.3 ± 0.0^**^	2.3 ± 0.0	3.3 ± 0.3^*^	−	−	−	−

Unpaired t-test

* *P ≤ 0.05*;

** *P ≤ 0.005*;

*** *P ≤ 0.001, indicates significant differences between the yeast concentration at 4 and 9 dpf*.

*ni, larvae not inoculated with yeasts*.

*<, < 1 (log_10_CFU/larva)*.

*−, Not determined*.

The initial concentration of *V. anguillarum* in CONV-R larvae at 5 dpf not treated with yeasts reached on average log_10_ 2.8 CFU/larva, and persisted with the same concentration at 9 dpf (Table 3). The pre-treatment with yeast Dh97 generally did not significantly affect the initial concentration of the pathogen for CONV-R larvae (ANOVA *P* > 0.05); however, unexpectedly the pre-treatment with Yl242 significantly enhanced the initial pathogen concentration compared to larvae not inoculated with yeasts (ANOVA *P* < 0.05; Table 3).

**Table 3:** **Initial counts (5 dpf) and persistence (9 dpf) of ***V. anguillarum*** in CONV-R and germ-free (GF) larvae**.

	***V. anguillarum*** **concentration (log**_10_**CFU/larva)**							
**Yeast dose**	**CONV-R** + **Dh97** + **Va**		**GF** + **Dh97** + **Va**		**CONV-R** + **Yl242** + **Va**		**GF** + **Yl242** + **Va**	
**log_10_CFU/mL**	**5 dpf**	**9 dpf**	**5 dpf**	**9 dpf**	**5 dpf**	**9 dpf**	**5 dpf**	**9 dpf**
ni	^a^2.8 ± 0.2	^a^2.9 ± 0.1	4.1 ± 0.1	4.3 ± 0.5	^a^2.8 ± 0.2	^a^2.9 ± 0.1	4.1 ± 0.1	4.3 ± 0.5
4	^a^2.9 ± 0.1	^b^2.1 ± 0.1^**^	−	−	^b^3.3 ± 0.3	^a^1.8 ± 0.0^**^	−	−
5	^a^2.6 ± 0.2	^b^2.0 ± 0.1^*^	−	−	^b^3.2 ± 0.1	^a^1.7 ± 0.1^***^	−	−
6	^b^3.2 ± 0.1	^b^2.0 ± 0.0^**^	2.3 ± 0.0	4.3 ± 0.1^***^	^b^3.2 ± 0.1	^a^3.4 ± 0.1	3.2 ± 0.0	4.1 ± 0.1^**^
7	^a^2.6 ± 0.0	^b^2.0 ± 0.4	−	−	^b^3.2 ± 0.4	^a^3.0 ± 0.0	−	−

Unpaired t-test

* *P ≤ 0.05*;

** *P ≤ 0.005*;

*** *P ≤ 0.001, indicates significant differences between the V. anguillarum concentration at 4 and 9 dpf. Letters indicate differences between treated and not inoculated larvae at the respective day, ANOVA with Dunnet multiple comparison corrected test*.

*ni, larvae not inoculated with yeasts*.

*−, Not determined*.

Comparing the pathogen load in CONV-R larvae at the end of the challenge (9 dpf) with the initial concentration (5 dpf), we observed that all concentrations of Dh97 significantly reduced the pathogen load (*P* < 0.05, unpaired *t*-test), except for the higher yeast doses (log_10_ 7 CFU/larva). However, comparing with non-yeast inoculated larvae all Dh97 doses were equally effective in reducing pathogen concentration at 9 dpf (Table 3; ANOVA *P* < 0.005). Similarly, pre-treatment with Yl242 reduced the pathogen load at 9 dpf compared to 5 dpf and compared to larvae not inoculated with yeast (*P* < 0.005, unpaired *t*-test). However, only yeast doses of log_10_ 4 and log_10_ 5 CFU/larva were effective.

When GF larvae were challenged with *V. anguillarum*, the initial pathogen concentration at 5 dpf reached significantly higher levels than in CONV-R larvae (*P* < 0.05, unpaired *t*-test). The pre-treatment of GF larvae with both yeasts significantly reduced the initial pathogen concentration (at 5 dpf), compared to GF-challenged larvae (*P* < 0.05, unpaired *t*-test). However, neither yeast avoided *V. anguillarum* growth in GF larvae; counts of the pathogen at 9 dpf reached a similar level to larvae not inoculated with yeasts. These results suggest that larval protection by yeasts against a *V. anguillarum* challenge is not only due to a reduction in the host pathogen load, and other mechanisms such as immune modulation may be involved.

### Innate immune response induced in larvae

To determine the role of yeast in immune modulation of the host we evaluated the relative expression of innate immune response marker genes in CONV-R and GF larvae challenged with *V. anguillarum*, including interleukin 1 beta (*il1b*), tumor necrosis factor a (*tnfa*), interleukin 10 (*il10*), complement component 3 (*c3*) and myeloid-specific peroxidase (*mpx*).

**Figure 2:**
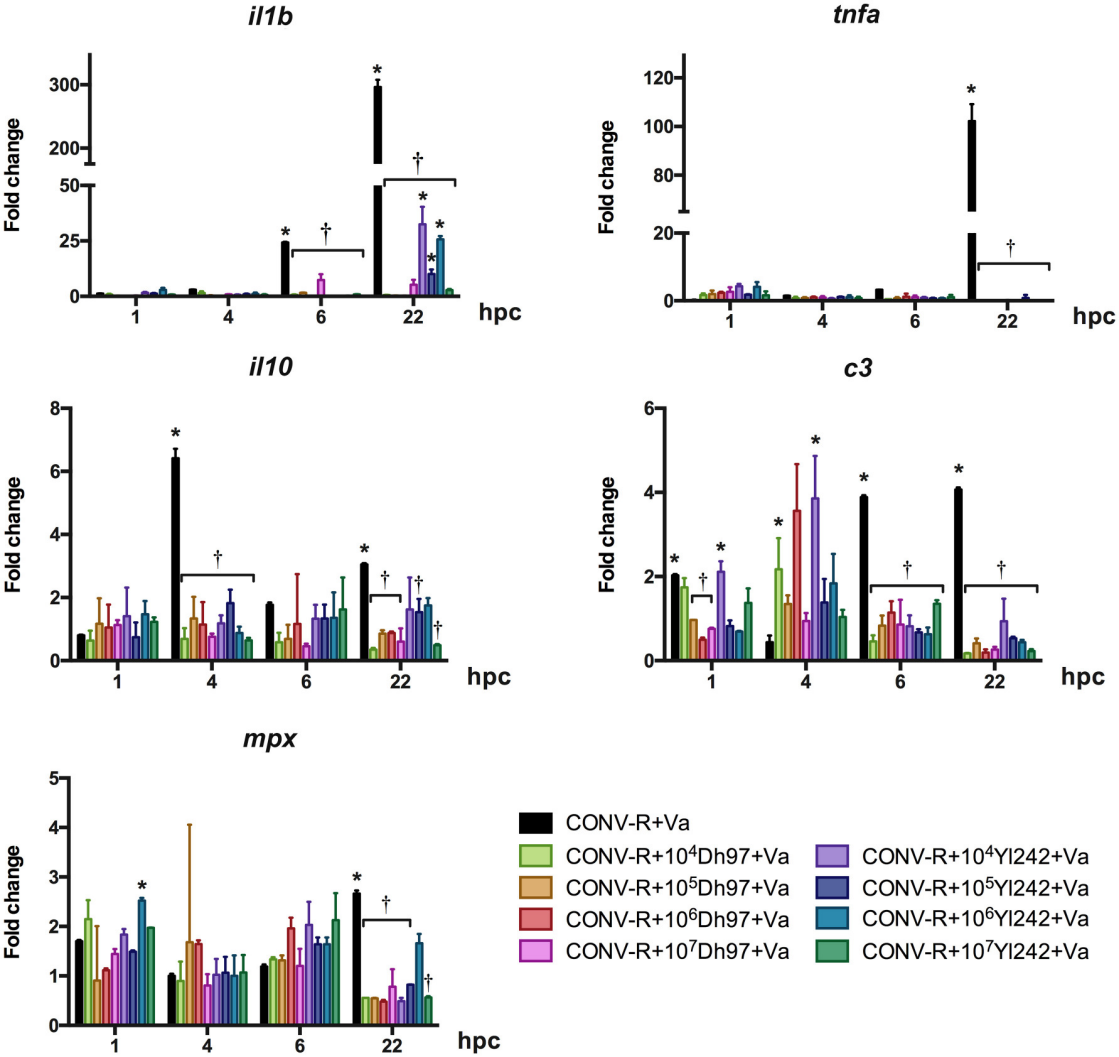
**Expression of innate immune genes analyzed by qPCR in conventionally raised (CONV-R) larvae challenged with ***V. anguillarum*** at 5 dpf, pre-treated at 4 dpf with different concentrations of each yeast, relative to non-challenged CONV-R larvae**. Dh97, *Debaryomyces hansenii* 97; Yl242, *Yarrowia lipolytica* 242; *il1b*, interleukin 1 beta; *tnfa*, tumor necrosis factor a; *c3*, complement component 3; *mpx*, myeloid-specific peroxidase; *il10*, interleukin 10; hpc, hours post *V. anguillarum*-challenge. Data were normalized to beta actin 1. The results show the mean ± SD of 3 independent experiments with three replicates each. ^*^Indicates statistically significant differences of the experimental groups with non-challenged CONV-R larvae, ^†^Indicates statistically significant differences of the *V. anguillarum*-challenged larvae treated with yeasts (CONV-R + yeast + Va) with *V. anguillarum*-challenged CONV-R larvae (CONV-R + Va).

CONV-R larvae challenged with *V. anguillarum* showed a significant upregulation of *il1b* at 6 and 22 h post-challenge (hpc), *c3* at 1, 6, and 22 h post-challenge (hpc) and *tnfa* and *mpx* at 22 hpc compared to un-challenged CONV-R larvae (Figure 2, Supplementary Table 1). The transcription level of the anti-inflammatory cytokine *il-10* was upregulated at 4 and 22 hpc. Interestingly, pre-treatment with yeast Dh97 or Yl242 significantly prevented the upregulation of all these genes (Figure 2, Supplementary Table 1). In general, all yeast doses were equally effective to prevent the upregulation of these genes (Figure 2, Supplementary Table 1).

**Figure 3:**
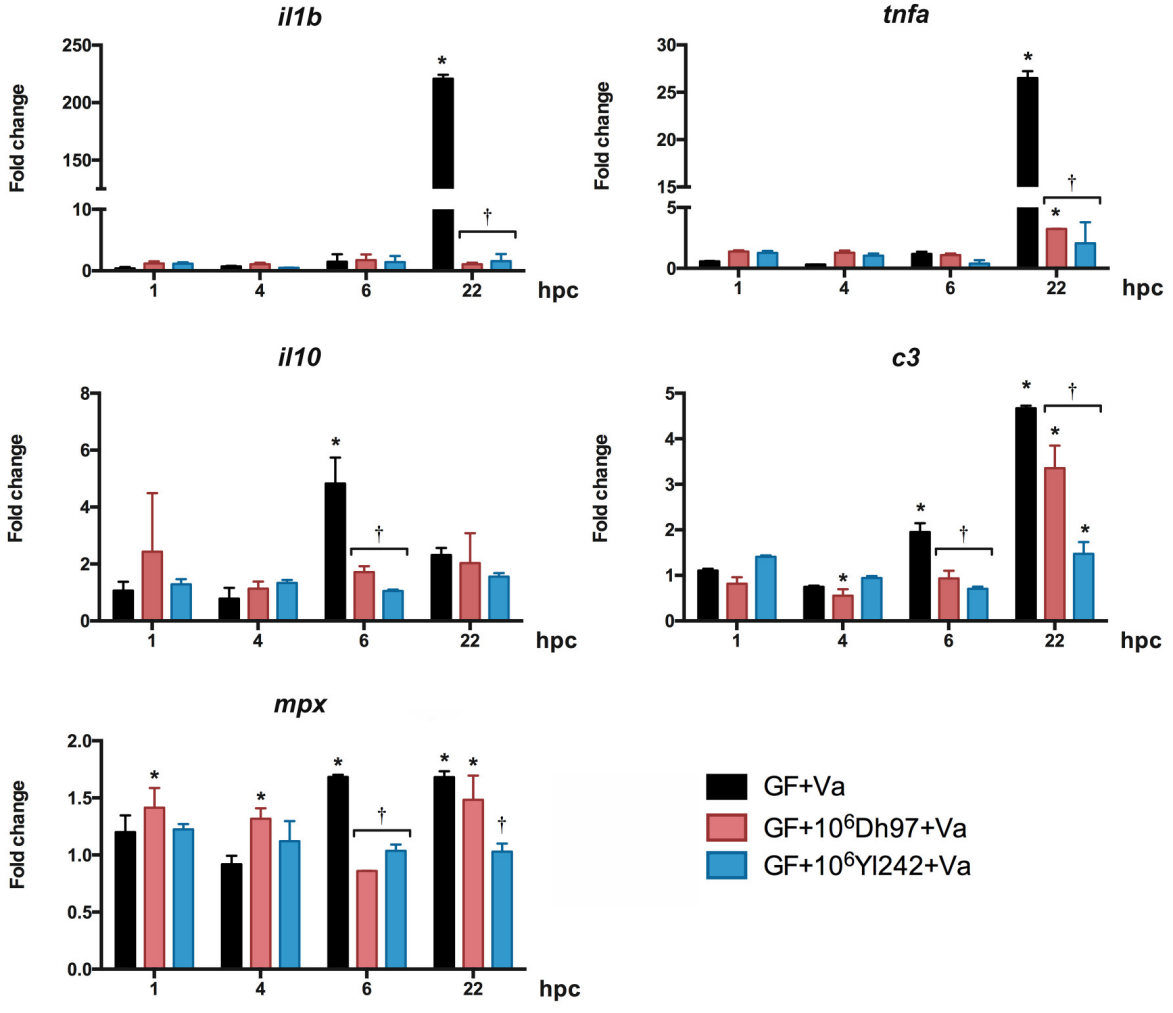
**Expression of innate-immune genes analyzed by qPCR in germ-free (GF) larvae challenged with ***V. anguillarum*** (Va) at 5 dpf, pre-treated at 4 dpf with 10^**6**^ CFU/mL of each yeast, relative to non-challenged germ-free larvae**. Dh97, *Debaryomyces hansenii* 97; Yl242, *Yarrowia lipolytica* 242; *il1b*, interleukin 1 beta; *tnfa*, tumor necrosis factor a; *c3*, complement component 3; *mpx*, myeloid-specific peroxidase; *il10*, interleukin 10; hpc, hours post *V. anguillarum* challenge. Data were normalized to beta actin 1. The results show the mean ± SD of 3 independent experiments with three replicates each. ^*^Indicates statistically significant differences of the experimental groups with non-challenged GF larvae, ^†^Indicates statistically significant differences between the *V. anguillarum*-challenged GF larvae treated with yeasts (GF + yeast + Va) with *V. anguillarum*-challenged GF larvae (GF + Va).

In GF larvae challenged with *V. anguillarum, il1b, tnfa*, and *c3* were significantly upregulated at 22 hpc, as in CONV-R larvae (Figure 3, Supplementary Table 2). *il10* was significantly upregulated at 6 hpc, and *mpx* was significantly upregulated at 6 and 22 hpc. Both yeasts, Dh97 and Yl242, significantly prevented the upregulation of *il1b, tnfa*, and *c3* at 22 hpc, and *il10* at 6 hpc (Figure 3, Supplementary Table 2).

To evaluate if yeasts alone could stimulate the innate immune system of larvae we measured the expression of the same genes in CONV-R and GF larvae treated with each yeast (Figures S3, S4, respectively). In CONV-R larvae (Figure S3 and Supplementary Table 3). *il1b* was upregulated by the two yeasts at 6 and 30 hpt (hour post-treatment), *tnfa* was upregulated by yeast Yl242 at concentrations of 10^4^ and 10^5^ (CFU/mL) at 46 hpt, *il10* was upregulated at 1, 6, 30, and 46 hpt by both yeasts, *c3* was upregulated only by some doses of yeast Dh97 at 6 and 24 hpt. Finally, *mpx* was only upregulated by Yl242 at 24 hpt at a dose of 10^6^ CFU/mL. None of the genes evaluated showed a dose-effect response. The overall gene expression induced by yeasts in GF larvae showed less change than in CONV-R (Figure S4 and Supplementary Table 4). Dh97 significantly downregulated the expression of *c3* at 1, 6, 22, 28, and 30 hpt and upregulated the expression of the gene at 46 hpt. On the other hand, yeast Yl242 upregulated *il1b* at 30 hpt, *tnfa* at 24 and 30 hpt, *il10* at 28 and 30 hpt, *c3* at 4, 24, 28, and 46 hpt, and *mpx* at 28, and 30 hpt, and downregulated *mpx* at 1 and 24 hpt.

### Co-aggregation studies

**Figure 4:**
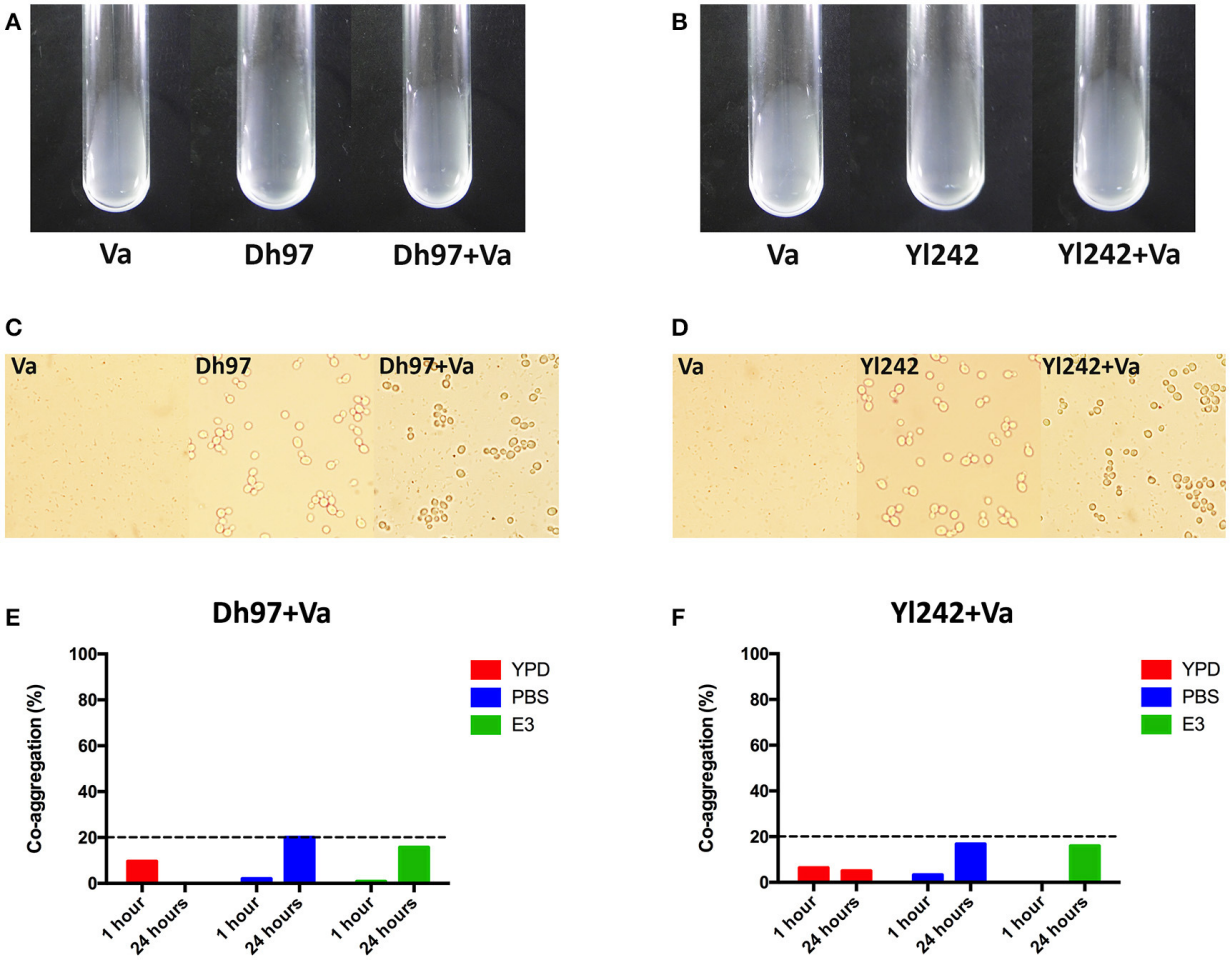
**Co-aggregation assays between the yeasts ***Debaryomyces hansenii*** (Dh97) or ***Yarrowia lipolytica*** (Yl242) with ***V. anguillarum*** (Va). (A,B)** Macroscopic co-aggregation assays. Equal volumes (200 μL) of *V. anguillarum* and **(A)** Dh97 or **(B)** Yl242 suspensions in co-aggregation buffer were mixed in borosilicate tubes (12 × 75 mm, Schott) for at least 5 s on vortex (Dh97 or Yl242 + Va tubes). Control tubes containing 200 μL of each microorganism and 200 μL of co-aggregation buffer (Va, Dh97, and Yl242 tubes) were included to check potential auto-aggregation. **(C,D)** Microscopic co-aggregation assays: all suspensions (from **A,B**) were observed in an optical microscope to observe any microscopic co-aggregation. **(E,F)** Spectrophotometric co-aggregation assays. A suspension of each yeast strain and *V. anguillarum* in YPD, PBS, and E3 (O.D. of 1.0 at 600 nm) were mixed in equal volumes (5 mL) for 10 s in vortex. Control tubes contained 10 mL of each microbial suspension. The O.D. (600 nm) of the upper suspension (1 mL) from each tubes was measured at 1 and 24 h. The percentage of co-aggregation was calculated as previously described (Ogunremi et al., 2015).

We determined if Dh97 and Yl242 yeasts could bind *V. anguillarum* through co-aggregation analysis. We did not detect any visual (Figures 4A,B) or microscopic auto- or co-aggregation (Figures 4C,D). We also performed a quantitative spectrophotometric co-aggregation assay using different media (YPD, PBS, or E3), since it has been reported that co-aggregation depends greatly on the conditions used (Millsap et al., 1998). All co-aggregation percentages were less than 20% in all media tested, showing no co-aggregation between the yeasts and *V. anguillarum* (Figures 4E,F).

## Discussion

The development of new probiotics requires not only *in vivo* demonstration of their benefits, but also an understanding of the mechanisms involved in their effects. In this study we explored some mechanisms involved in the protection of zebrafish larvae against a *V. anguillarum* challenge by two probiotic yeasts, *D. hansenii* 97 (Dh97) and *Y. lipolytica* 242 (Yl242), isolated from the intestine of healthy fish (Raggi et al., 2014). We analyzed the effect of both yeasts on *in vivo* pathogen concentration, modulation of the host innate immune system and co-aggregation with the pathogen. In addition, the effect of zebrafish microbiota on the survival of larvae was determined using germ-free (GF) larvae.

As previously reported (Caruffo et al., 2015), challenging CONV-R zebrafish larvae with *V. anguillarum* provokes high mortality (>60%) at 4 days post-challenge (dpc). Studies performed in mouse models have shown that an intact microbiota protects the host against pathogen attack (Endt et al., 2010). To determine the potential protective role of the zebrafish microbiota against *V. anguillarum* challenge, GF larvae were exposed to the pathogen. Our results show, for the first time in zebrafish, that the resident microbiota can protect the host from *V. anguillarum* infection, since GF animals showed significantly more mortality (92%) than CONV-R larvae. In opposition to our results, no protective role of the resident zebrafish microbiota was detected in larvae exposed to the same pathogen (Oyarbide et al., 2015) or *Edwarsiella ictaluri* (Rendueles et al., 2012). Similarly, no protective effect of the host microbiota was observed in a novel infection model of gnotobiotic Nile tilapia with *E. ictaluri* (Situmorang et al., 2014). These results suggest that the specific composition of the resident microbiota of zebrafish in our facilities may be more effective in protecting larvae against *V. anguillarum* infection. Future studies should characterize the composition of this microbiota to elucidate the specific microorganisms involved in protection.

We then explored the capacity of two yeast strains isolated from healthy fish to protect zebrafish larvae from *V. anguillarum* challenge. In conventionalized conditions at a dose of 10^6^ CFU/mL, yeast Dh97 was more effective than Yl242 in protecting the larvae. The different colonization capacity of the yeasts in CONV-R larvae may explain this difference. In germ-free conditions the yeasts showed similar colonization capacities and were equally effective against the pathogen, and surprisingly, more effective than the host microbiota to protect larvae. Overall, the results suggest that yeast concentrations inside larvae were more determinant than yeast proliferation to protect them from a unique dose of the pathogen. In addition, these results showed that protection exerted by yeasts is not necessarily related to the modulation of the host microbiota.

The concentration of the pathogen in CONV-R larvae at the moment of the challenge and at the end of the experiment were similar (*P* > 0.05), and reached about log_10_ 2.8 CFU/larva. In a similar study (Oyarbide et al., 2015) with larvae exposed to *V. anguillarum*, the pathogen reached higher concentrations (log_10_ 5.9 and 5.8 per larvae at 5 and 6 dpf, respectively) and produced higher mortality (100% after 3 days post-*V. anguillarum* challenge at 8 dpf) than in our study. This result could be due to differences in the virulence of the strains, differences in the susceptibility of the hosts, higher concentration of *V. anguillarum* inoculated or to the design of the experiment, in which larvae were constantly exposed to the pathogen (Oyarbide et al., 2015), in contrast with our study. The reduced survival observed in challenged GF larvae during our experiments could be related to higher concentrations of the pathogen in larvae (>1 log) than those observed for challenged CONV-R larvae and/or due to the lack of host microbiota protection.

We then evaluated the capacity of the yeasts to reduce the pathogen concentration in *V. anguillarum*-challenged larvae in CONV-R and GF conditions. The two yeasts modified pathogen concentrations during the challenge. The initial concentration of the pathogen reached in CONV-R larvae was not modified, except at the higher doses of Yl242, which increased the initial concentration of the pathogen, suggesting that this yeast could stimulate *V. anguillarum* entrance to the host. The possible mechanisms explaining this point could involve greater habitat availability inside the gut by eventual modification of the gut microbiota by Yl242, or modification of intestinal mucus layer, enhancing the chemotactic swimming of *V. anguillarum* toward the intestinal mucus (O'Toole et al., 1999), although these hypotheses merit more study. At the end of the challenge both yeasts tended to reduce the pathogen concentration in CONV-R larvae. In germ-free larvae, this anti-bacterial effect was only observed at the beginning of the challenge, but yeasts were unable to control pathogen growth; the pathogen reached the same concentration at 9 dpf as in germ-free larvae not treated with yeasts. These results contrast with those obtained in yeast-treated CONV-R larvae, where yeasts tended to reduce the bacterial load at 9 dpf. This difference is probably due to an indirect effect of the yeast on the host microbiota, because reduction of pathogen growth was not observed in germ-free larvae. Surprisingly, in spite of the higher *V. anguillarum* concentration, in germ-free larvae the survival of yeast-treated larvae was equivalent to yeast-treated CONV-R larvae. We hypothesize that yeasts could exert other mechanisms to reduce the virulence of *V. anguillarum* that could explain lower mortality observed. These findings are in accordance with a previous study showing that protection of *S. boulardii* against *Salmonella* infection in mice is not related to *in vivo* antagonism (Martins et al., 2013). Overall, these results showed that larval protection by yeasts is not always associated with an *in vivo* anti-pathogen effect, as previously described (Schneider, 2011). These results suggest that other mechanisms besides the control of pathogen replication may be involved in protection, such as modulation of the immune response of the host.

The inflammatory signaling cascade is triggered when the host receptors involved are capable of binding to the bacteria or their products. This process results in the production of several pro-inflammatory cytokines such as *il1b* and *tnfa* (van der Vaart et al., 2012). A strong inflammatory response in larvae was observed after the *V. anguillarum* challenge, reflected by a robust upregulation at the transcriptional level of *tnfa* and *il1b*, as previously described in zebrafish larvae infected with *V. anguillarum* (Oyarbide et al., 2015) and *E. ictaluri* (Rendueles et al., 2012). The challenged larvae also exhibited an up-regulation of the mRNA level of *c3, mpx* and *il10*. *c3* is the best characterized component of the complement system; it plays a central role in all activation pathways (Lee et al., 2013) and it is crucial in the early immune response of fish larvae (Løvoll et al., 2007). Its expression is induced by LPS, and in zebrafish it plays a role in inflammatory processes and regeneration (Forn-Cuní et al., 2014). *mpx* is one of the most specific markers for neutrophil and its precursors. Its expression is related to myelopoiesis (Bennett et al., 2001; Glenn et al., 2014). The upregulation of this gene in challenged larvae could reflect active neutrophil proliferation derived from the inflammatory response induced by the pathogen. *il10* targets various leukocytes and mainly represses or modulates excessive inflammatory responses (Ouyang et al., 2011). The induction of this cytokine reveals a modulatory response of the host to the induced inflammation triggered by the pathogen. Importantly, in our study the analysis of cytokine expression was performed only until 22 h post-*V. anguillarum* challenge, since previous reports have shown that most of the transcripts are modulated in the first 24 h after *V. anguillarum* infection (Rojo et al., 2007; Zhang et al., 2013; Liu et al., 2014; Oyarbide et al., 2015). Previous results showed a significant increase of *tnfa, il1b*, and *il10* over time in zebrafish larvae infected by *E. ictaluri* up to 3 days post-infection (Rendueles et al., 2012). In our study, it would be important to evaluate the immune modulation exerted by *V. anguillarum* until the end of the trial (4 dpc), to determine its correlation with larval mortality.

The pre-treatment of CONV-R and germ-free larvae with yeasts completely prevented upregulation of all immune relevant genes evaluated at 22 hpc. It has been previously described that yeasts can also show anti-inflammatory effects. The yeast *S. cerevisiae var. boulardii* can modulate the immune system response during bacterial infection (Czerucka et al., 2007; Moslehi-Jenabian et al., 2010). This yeast can exert anti-inflammatory effects related to the suppression of NF-κB activation, inhibition of the pro-inflammatory cytokine gene expression and stimulation of PPAR-γ expression, reducing enterocyte responses to pro-inflammatory cytokines. Whether, these mechanisms could be involved in larval protection merits further analysis.

It has been widely described that neutrophil migration in zebrafish larvae, considered a key hallmark in an inflammatory process, is correlated with the expression of some inflammatory cytokines such as *tnfa* and *il1b* (Barros-Becker et al., 2012; Hedrera et al., 2013; de Oliveira et al., 2016). In a previous study (Caruffo et al., 2015) we observed an increase in neutrophil migration outside the hematopoietic region at 3 hpc in CONV-R larvae challenged with *V. anguillarum*, showing an inflammatory response of the host. Although, in the present study we did not evaluate neutrophil migration, we would expect an increase in neutrophil migration outside the hematopoietic tissue during all the infection period with *V. anguillarum* in CONV-R and germ-free larvae, concomitant with a reduced number of inflammatory cells in larvae pre-treated with yeasts.

Yeasts contain β-glucans, mannoproteins, and chitin in their cell walls, and also nucleotides which can stimulate the immune system by binding to specific receptors (Reyes-Becerril et al., 2008; Oyarbide et al., 2012; Barreto-Bergter and Figueiredo, 2014). The immuno-stimulatory effect of yeast β-glucans, which are part of the pathogen-associated molecular patterns (PAMPS), is well-known and has proven to be efficient in different fish species (Bricknell and Dalmo, 2005; Magnadottir, 2010) including zebrafish (Rodríguez et al., 2009). β-glucans are located on the inner cell wall layer of yeasts, protected by an outer layer of mannoproteins (Erwing and Gow, 2016). It has been described that the immune effect of β-glucans depends on their structure and the level of exposure of these molecules to the host immune cells (Navarrete and Tovar-Ramírez, 2014; Erwing and Gow, 2016). For example, juvenile rainbow trout (*O. mykiss*) fed with a beta-mercaptoethanol-treated *S. cerevisiae*-supplemented diet (with an expected more open structure of the yeast cell wall due to the breaks of the disulfide bonds between mannoproteins) showed higher stimulation of the immune system and an enhanced survival rate against *Yersinia ruckeri* compared to fish fed with whole-cell yeast (Tukmechi et al., 2011). It is noteworthy that all studies have been performed with β-glucans derived from *S. cerevisiae*, and little is known about the immunomodulatory effect of β-glucans derived from non-*S. cerevisiae* yeasts. It is known that yeast species have different cell wall composition, with different proportions of glucans (Nguyen et al., 1998), suggesting that they can differentially modulate the host immune system. This difference could explain, in part, the different immune modulations observed with Dh97 and Yl242, or the different protection magnitude by different yeast species in a *V. anguillarum* challenge model (Caruffo et al., 2015).

Previous work showed that immune stimulation by yeast in gilthead seabream (*Sparus aurata* L.) displays increased or decreased expression of the immune genes according to the organ evaluated (intestine, head kidney, and liver; Reyes-Becerril et al., 2008). In this study we tested the stimulation of the innate immune system in larvae treated with yeasts. Although we did not observe a time-, dose-, or yeast-specific response, yeasts were able to modulate some of the genes evaluated. The lack of a clear tendency in cytokine expression could be explained because we evaluated the transcripts in the entire larvae and not in each organ. The magnitude of cytokine expression induced by both yeasts in non-*V. anguillarum* challenged larvae was lower than in those stimulated by the pathogen. This could be related to the point discussed above, i.e., to the grade of exposure of immune-stimulating molecules in the cell wall of these two yeasts, or because the interaction of *V. anguillarum* with the host is greater due to the invasive nature of this pathogen. Related to the last point, it has been reported that larvae challenged with GFP-labeled *V. anguillarum* harbor the pathogen in the gastrointestinal tract at 3 hpc (O'Toole et al., 2004), as we previously observed (Caruffo et al., 2015), and in the head and tail after 6 hpc (O'Toole et al., 2004). By contrast, five probiotic yeast candidates including Yl242 were only observed in the gastrointestinal tract 5 days after yeast treatment (Caruffo et al., 2015).

In our study both yeasts were able to remain viable in larvae until the end of the *V. anguillarum* challenge. However, we do not know if protection against this pathogen or immune modulation needs viable yeast. This point is essential to a better understanding of the mechanisms involved in yeast protection. One would expect that protection mechanisms by dead yeasts could include competition for the physical space (in the gut), stimulation of the immune system by their cell wall components, adhesion to the pathogen impeding its invasion of the host (Moslehi-Jenabian et al., 2010) or promoting its elimination by feces (Pontier-Bres et al., 2014). On the other hand, live yeasts could also contribute with secreted factors (Ran et al., 2016). The importance of yeast viability on the probiotic effect has been recently studied and shows that the effect is influenced by fish density (Ran et al., 2015, 2016). Under high stocking density, supplementation of live *S. cerevisiae* in the feed of Nile tilapia significantly enhanced resistance of fish against *Aeromonas hydrophila* compared to heat-inactivated yeast (Ran et al., 2016). However, under normal fish density both live and inactivated yeast protected the host against infection by *A. hydrophila*. In addition, live yeast, but not inactivated yeast, reduced intestinal expression of *tnf*α, *tgf*β, and *il1*β (Ran et al., 2015), showing the importance of secreted factors in the host immune modulation. In our study, it seems that multiple species-specific mechanisms are involved in protection against *V. anguillarum*. Future studies including protection experiments using dead yeast (i.e., heat-inactivated yeast) will help to elucidate this issue.

It has been reported that *S. cerevisiae* var. *boulardii* can prevent the adherence and translocation of bacteria to enterocytes, which can be explained in part by their ability to bind bacteria (Moslehi-Jenabian et al., 2010). Because yeast treatments completely abolish the inflammatory response induced by *V. anguillarum*, we tested the hypothesis that yeasts could adhere to *V. anguillarum*, impeding or reducing its contact with the host, which would explain in part the protective role of yeasts. These experiments were performed with *in vitro* co-aggregation assays. Co-aggregation has been defined as a specific recognition and adhesion of genetically distinct bacteria when they are in suspension, which is mediated by adhesins and polysaccharide receptors on the cell surface of co-aggregating cells (Kolenbrander, 2000; Rickard et al., 2003; Vornhagen et al., 2013). This specific interaction has been observed in human intestinal bacteria (Ledder et al., 2008), and recently between yeast and bacteria (Martins et al., 2013; Stevens et al., 2015). We did not observe any *in vitro* co-aggregation between yeasts and *V. anguillarum* in any of the assays performed. A previous study showed *in vivo* binding of the yeast *S. cerevisiae* (UFMG 905) with *S. enterica* serovar Typhimurium, reducing its translocation and invasion in mice (Martins et al., 2013). Whether this yeast-*V. anguillarum* interaction could occur *in vivo* requires further investigation.

In addition to the mechanisms evaluated in this study, yeasts can protect the host from pathogens via other pathways, mainly in the gut. For example, yeasts can improve the intestinal barrier function, stabilizing tight junctions and reducing pathogen translocation (Moslehi-Jenabian et al., 2010). The trophic effect of yeasts due to the production of polyamines (mainly spermine and spermidine) has been well described in humans, rodents and fish (Tovar-Ramírez et al., 2004; Buts and De Keyser, 2006). Although, this trophic effect has not been evaluated in the protection against a pathogen challenge, it could be postulated that this mechanism may also improve host survival. Recently, yeasts have also been shown to affect the intestinal traffic of the pathogen *Salmonella* Typhimurium. The adhesion of the pathogen and the yeast modifies pathogen distribution in the lumen, increasing its elimination in feces (Pontier-Bres et al., 2014). Further studies are necessary to elucidate if these mechanisms are also involved in the protection of zebrafish larvae against the *V. anguillarum* challenge by yeasts Dh97 and Yl242.

In conclusion, our results revealed that protection of zebrafish larvae against a *V. anguillarum* challenge with two non-*Saccharomyces* yeasts involves strain-specific mechanisms. Yeasts were able to modulate the innate immune system of the host and showed an *in vivo* anti-pathogen effect; however, the lower mortality with yeast pretreatment does not always correlate with lower pathogen burden. This suggests that other protection mechanisms may be involved. In addition, using GF larvae we highlighted the importance of the normal resident microbiota to enhance the host response to a bacterial infection, and showed the utility of using probiotic yeasts to restore or even improve the beneficial effect exerted by the host microbiota. Whether the beneficial effects of yeasts include other mechanisms will be explored in future investigations. Thus, our results provide new insight into the complex microbial interaction between a beneficial and pathogenic microorganisms and the host in the context of health and disease.

## Author contributions

MC, NN, and PN conceived and designed the experiments. MC, NN, OS performed the experiments. MG, NF performed the co-aggregation experiments. MC, NN, KG, CF, AR, and PN analyzed the data. MC, PN wrote the paper.

## Funding

This work was supported by FONDECYT N°11110414. MC acknowledges a scholarship from CONICYT N°21110848 and Dr. Stekel fellowship, INTA-Nestlé.

### Conflict of interest statement

The authors declare that the research was conducted in the absence of any commercial or financial relationships that could be construed as a potential conflict of interest.
