# Supplementary material for: Protective Yeasts Control V. anguillarum Pathogenicity and Modulate the Innate Immune Response of Challenged Zebrafish (Danio rerio) Larvae
Source: Front Cell Infect Microbiol. 2016 Oct 14;6:127. doi: 10.3389/fcimb.2016.00127 (PMC5063852; doi:10.3389/fcimb.2016.00127)
Supplement: Supplementary file 2 [file Table2.docx]

**Supplementary Table 2.** **Gene expression analysis treatment comparisons.**

Differences between treatments were analyzed by ANOVA with Newman-Keuls test.

|  |  |  |  |  |  |  |  |  |  |
| --- | --- | --- | --- | --- | --- | --- | --- | --- | --- |
| **Gen** | **Treatment comparison** | **1 hpt** | **4 hpt** | **6 hpt** | **22 hpt** | **24 hpt** | **28 hpt** | **30 hpt** | **46 hpt** |
| *il1b* | CONV-R vs. CONV-R 10^4 Dh97 | ns | ns | **** | ns | ns | ns | ns | ns |
| *il1b* | CONV-R vs. CONV-R 10^5 Dh97 | ns | ns | ns | ns | ns | ns | ns | ns |
| *il1b* | CONV-R vs. CONV-R 10^6 Dh97 | ns | ns | ns | ns | ns | ns | ns | ns |
| *il1b* | CONV-R vs. CONV-R 10^7 Dh97 | ns | ns | **** | ns | ns | ns | * | ns |
| *il1b* | CONV-R vs. CONV-R 10^4 Yl242 | ns | ns | ns | ns | ns | ns | ns | ns |
| *il1b* | CONV-R vs. CONV-R 10^5 Yl242 | ns | ns | ns | ns | ns | ns | ns | ns |
| *il1b* | CONV-R vs. CONV-R 10^6 Yl242 | ns | ns | ns | ns | ns | ns | ** | ns |
| *il1b* | CONV-R vs. CONV-R 10^7 Yl242 | ns | ns | **** | ns | ns | ns | ns | ns |
| *tnfa* | CONV-R vs. CONV-R 10^4 Dh97 | ns | ns | ns | ns | ns | ns | ns | ns |
| *tnfa* | CONV-R vs. CONV-R 10^5 Dh97 | ns | ns | ns | ns | ns | ns | ns | ns |
| *tnfa* | CONV-R vs. CONV-R 10^6 Dh97 | ns | ns | ns | ns | ns | ns | ns | ns |
| *tnfa* | CONV-R vs. CONV-R 10^7 Dh97 | ns | ns | ns | ns | ns | ns | ns | ns |
| *tnfa* | CONV-R vs. CONV-R 10^4 Yl242 | ns | ns | ns | ns | ns | ns | ns | *** |
| *tnfa* | CONV-R vs. CONV-R 10^5 Yl242 | ns | ns | ns | ns | ns | ns | ns | *** |
| *tnfa* | CONV-R vs. CONV-R 10^6 Yl242 | ns | ns | ns | ns | ns | ns | ns | ns |
| *tnfa* | CONV-R vs. CONV-R 10^7 Yl242 | ns | ns | ns | ns | ns | ns | ns | ns |
| *c3* | CONV-R vs. CONV-R 10^4 Dh97 | ns | ns | *** | ns | ns | ns | ns | ns |
| *c3* | CONV-R vs. CONV-R 10^5 Dh97 | ns | ns | ns | ns | ns | ns | ns | ns |
| *c3* | CONV-R vs. CONV-R 10^6 Dh97 | ns | ns | ns | ns | * | ns | ns | ns |
| *c3* | CONV-R vs. CONV-R 10^7 Dh97 | ns | ns | * | ns | ns | ns | ns | ns |
| *c3* | CONV-R vs. CONV-R 10^4 Yl242 | ns | ns | ns | ns | ns | ns | ns | ns |
| *c3* | CONV-R vs. CONV-R 10^5 Yl242 | ns | ns | ns | ns | ns | ns | ns | ns |
| *c3* | CONV-R vs. CONV-R 10^6 Yl242 | ns | ns | ns | ns | ns | ns | ns | ns |
| *c3* | CONV-R vs. CONV-R 10^7 Yl242 | ns | ns | ns | ns | ns | ns | ns | ns |
| *il10* | CONV-R vs. CONV-R 10^4 Dh97 | ns | ns | ** | ns | ns | ns | ns | ns |
| *il10* | CONV-R vs. CONV-R 10^5 Dh97 | ns | ns | ns | ns | ns | ns | ** | ns |
| *il10* | CONV-R vs. CONV-R 10^6 Dh97 | ns | ns | ns | ns | ns | ns | ns | ns |
| *il10* | CONV-R vs. CONV-R 10^7 Dh97 | **** | ns | * | ns | ns | ns | ** | ns |
| *il10* | CONV-R vs. CONV-R 10^4 Yl242 | ns | ns | ns | ns | ns | ns | ns | ** |
| *il10* | CONV-R vs. CONV-R 10^5 Yl242 | ns | ns | ns | ns | ns | ns | ns | ns |
| *il10* | CONV-R vs. CONV-R 10^6 Yl242 | ns | ns | ns | ns | ns | ns | ns | ns |
| *il10* | CONV-R vs. CONV-R 10^7 Yl242 | ns | ns | * | ns | ns | ns | ns | ns |
| *mpx* | CONV-R vs. CONV-R 10^4 Dh97 | ns | ns | ns | ns | ns | ns | ns | ns |
| *mpx* | CONV-R vs. CONV-R 10^5 Dh97 | ns | ns | ns | ns | ns | ns | ns | ns |
| *mpx* | CONV-R vs. CONV-R 10^6 Dh97 | ns | ns | ns | ns | ns | ns | ns | ns |
| *mpx* | CONV-R vs. CONV-R 10^7 Dh97 | ns | ns | ns | ns | ns | ns | ns | ns |
| *mpx* | CONV-R vs. CONV-R 10^4 Yl242 | ns | ns | ns | ns | ns | ns | ns | ns |
| *mpx* | CONV-R vs. CONV-R 10^5 Yl242 | ns | ns | ns | ns | ns | ns | ns | ns |
| *mpx* | CONV-R vs. CONV-R 10^6 Yl242 | ns | ns | ns | ns | **** | ns | ns | ns |
| *mpx* | CONV-R vs. CONV-R 10^7 Yl242 | ns | ns | ns | ns | ns | ns | ns | ns |

* P ≤ 0.05; ** P ≤ 0.005; *** P ≤ 0.001; **** P ≤ 0.0001, indicates significant differences between the treatment comparisons.

ns: not significant
