# Supplementary material for: Protective Yeasts Control V. anguillarum Pathogenicity and Modulate the Innate Immune Response of Challenged Zebrafish (Danio rerio) Larvae
Source: Front Cell Infect Microbiol. 2016 Oct 14;6:127. doi: 10.3389/fcimb.2016.00127 (PMC5063852; doi:10.3389/fcimb.2016.00127)
Supplement: Supplementary file 5 [file Image1.pdf]

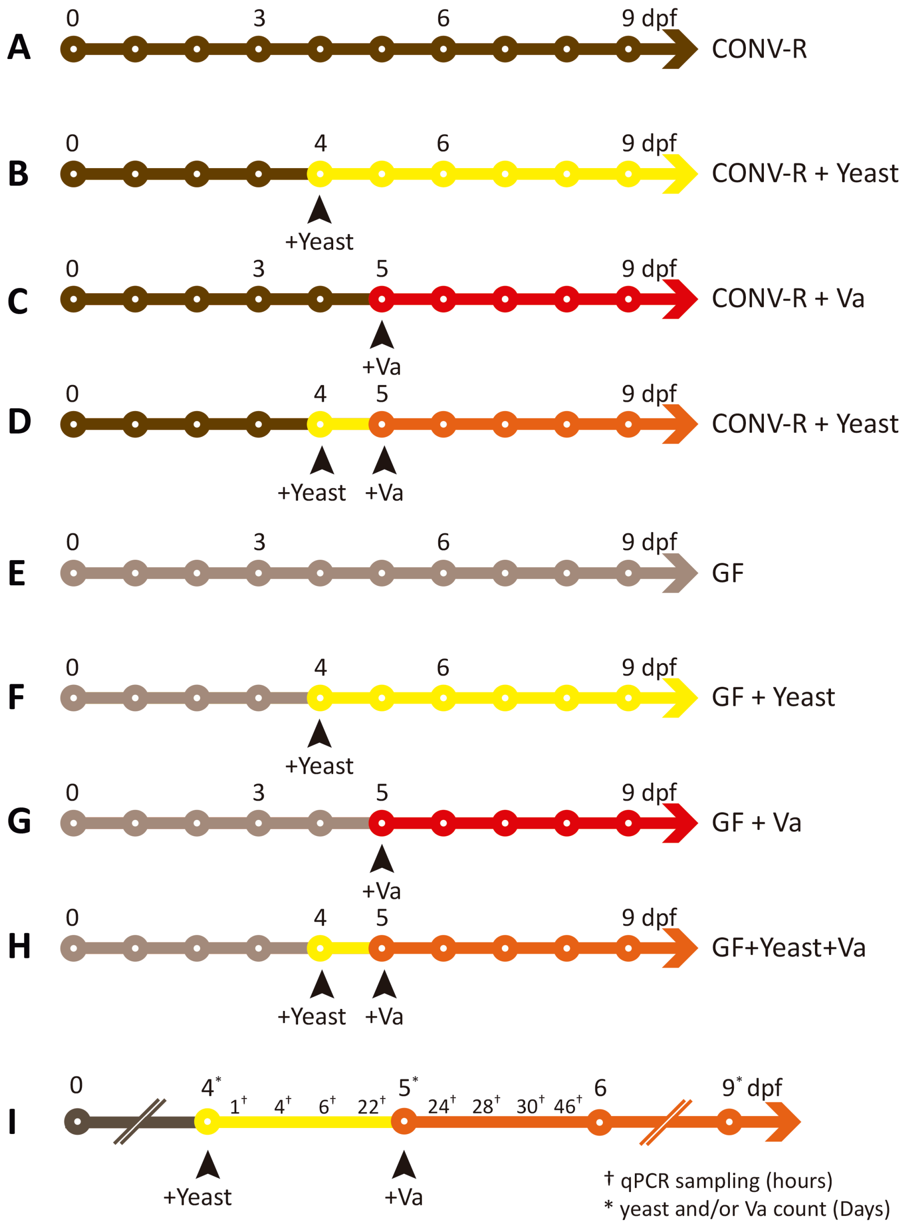

Figure S1. Scheme of the different treatments used and sampling points for Va/yeast counts and qPCR. A: CONV-R, conventionally raised larvae; B: CONV-R + Yeast: conventionally raised larvae and inoculated with yeast at 4 days post fertilization (dpf); C: CONV-R + Va: conventionally raised larvae and challenged at 5 dpf with *V. anguillarum*; D: CONV-R + Yeast + Va: conventionally raised larvae, inoculated with yeast at 4 dpf and challenged with *V. anguillarum* at 5 dpf; E: GF, germ free larvae; F: GF + Yeast: germ free larvae inoculated with yeast at 4 dpf; G: GF + Va, germ free larvae challenged with *V. anguillarum* at 5 dpf; H: germ free larvae inoculated with yeast at 4 dpf and challenged with *V. anguillarum* at 5 dpf; I: sampling scheme for qPCR analysis† and yeast and *V. anguillarum* counts*.
