# Supplementary material for: Protective Yeasts Control V. anguillarum Pathogenicity and Modulate the Innate Immune Response of Challenged Zebrafish (Danio rerio) Larvae
Source: Front Cell Infect Microbiol. 2016 Oct 14;6:127. doi: 10.3389/fcimb.2016.00127 (PMC5063852; doi:10.3389/fcimb.2016.00127)
Supplement: Supplementary file 6 [file Image2.pdf]

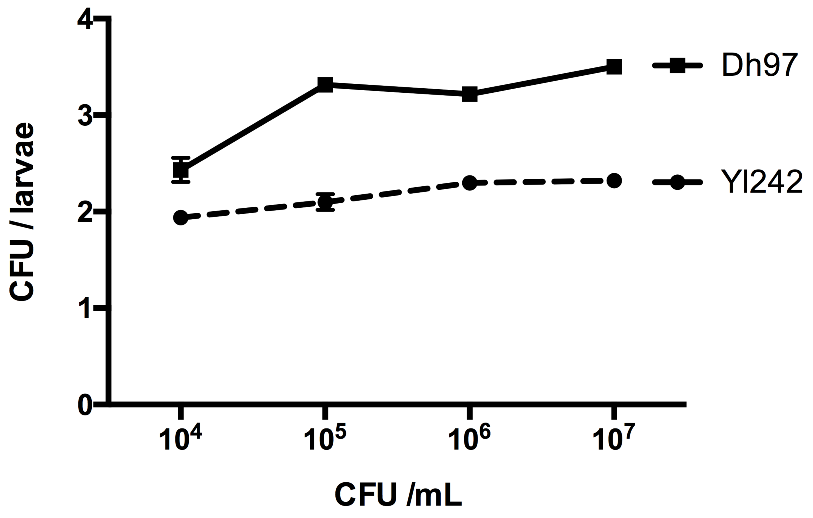

Figure S2. Initial concentration of yeasts at 4 days post fertilization (dpf) after 2h immersion with yeasts *Debaryomyces hansenii* (Dh97) and *Yarrowia lypolytica* (Yl242). Dh97 reached higher concentrations in larvae than Yl242 ($P<0.05$, unpaired t test). The two yeasts showed a positive correlation between yeast inoculum and yeast concentration in larvae ($r=0.9203$, $P<0.0001$ for Dh97; and $r=0.7778$, $P<0.001$ for Yl242, Spearman correlation test).
