# Supplementary material for: Protective Yeasts Control V. anguillarum Pathogenicity and Modulate the Innate Immune Response of Challenged Zebrafish (Danio rerio) Larvae
Source: Front Cell Infect Microbiol. 2016 Oct 14;6:127. doi: 10.3389/fcimb.2016.00127 (PMC5063852; doi:10.3389/fcimb.2016.00127)
Supplement: Supplementary file 8 [file Image4.PDF]

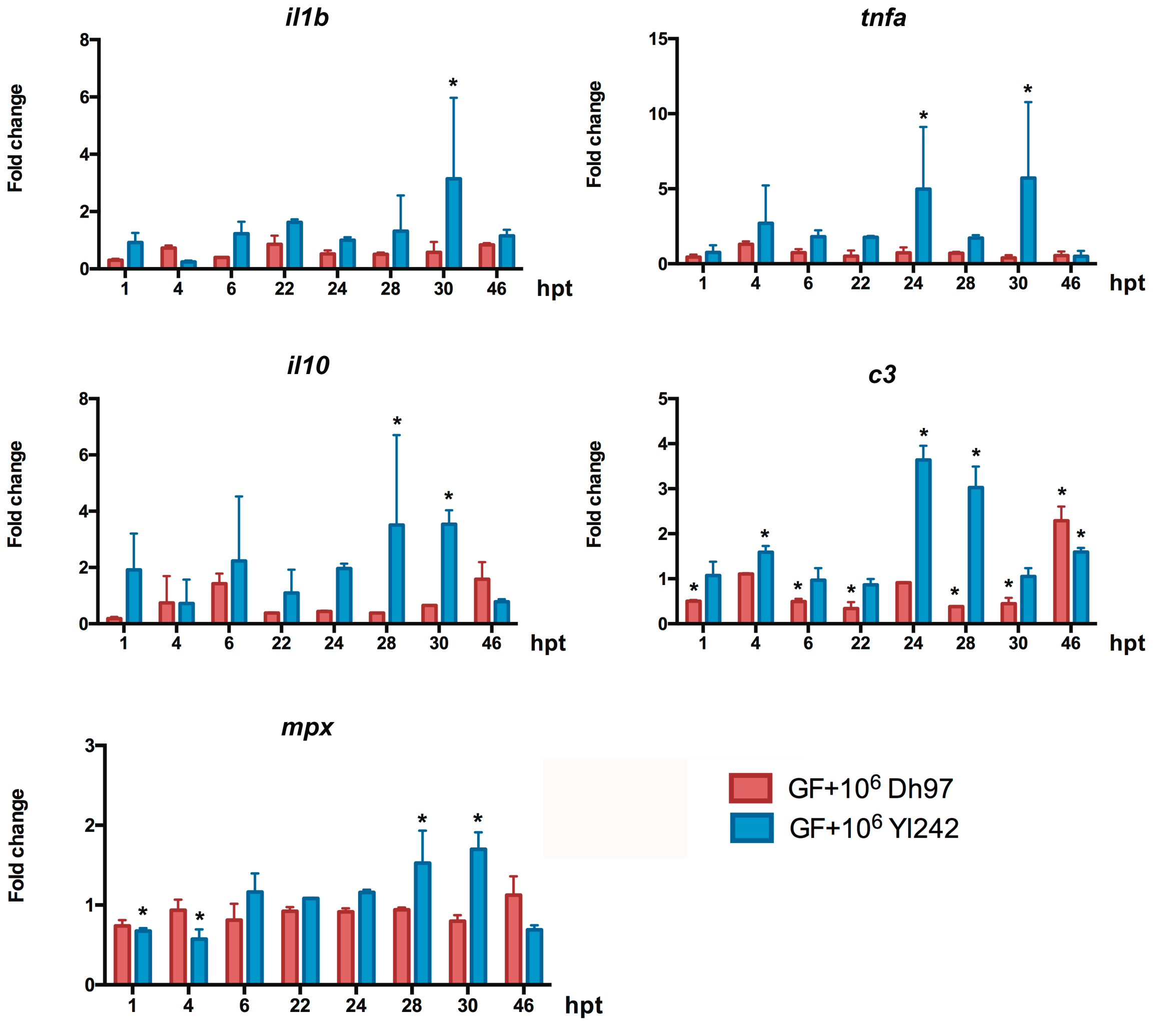

Figure S4: Expression of innate-immune genes analyzed by qPCR in germ free (GF) larvae treated at 4 dpf with 10^6 CFU/mL of each yeast, relative to non-treated GF larvae. Dh97: *Debaryomyces hansenii* 97, Yl242: *Yarrowia lipolytica* 242, *il1b*: interleukin 1 beta, *tnfa*: tumor necrosis factor a, *c3*: complement component 3, *mpx*: myeloid-specific peroxidase, *il10*: interleukin 10, hpt: hours post-treatment. Data were normalized to beta actin 1. The results show the mean ± SD of 3 independent experiments with three replicates each time. *: indicates statistically significant differences of the experimental groups with non-treated GF larvae.
